# Clinical Application of Minimally Invasive Punch and Drainage in the Treatment of Refractory Non-purulent Skin and Soft Tissue Infections: A Case Series

**DOI:** 10.7759/cureus.102335

**Published:** 2026-01-26

**Authors:** Kevin Kuan-I Lee, Yun-Nan Lin

**Affiliations:** 1 Division of Hand Surgery, Department of Orthopedic Surgery, NYU Langone Health, New York, USA; 2 Division of Plastic Surgery, Department of Surgery, Kaohsiung Medical University Hospital, Kaohsiung, TWN

**Keywords:** inflammatory substance, minimally invasive, non-purulent, punch and drainage, skin and soft tissue infections

## Abstract

Non-purulent skin and soft tissue infections (NP-SSTIs) are commonly managed with antibiotic therapy; however, a subset of patients demonstrate inadequate clinical response and develop refractory disease. At present, no standardized minimally invasive procedural intervention exists for the management of refractory NP-SSTIs, and treatment options are particularly limited in elderly patients with multiple comorbidities. We report four cases evaluating a minimally invasive punch and drainage approach in elderly patients with persistent NP-SSTIs that failed to improve despite appropriate antibiotic therapy. These individuals had a mean age of 86.8 years (range, 75-99 years) and presented with ongoing local inflammation, pain, and systemic inflammatory responses accompanied by elevated inflammatory markers. Following unsuccessful antibiotic treatment, all patients underwent a minimally invasive punch and drainage procedure, in which circular punch incisions were created to facilitate drainage of inflammatory material and promote local infection control. Standard postoperative wound care and follow-up were provided. Clinical improvement and effective infection control were observed in all cases without significant perioperative or postoperative complications. Patients were discharged between 7 and 42 days after the procedure. The mean C-reactive protein level decreased from 134.52 mg/L preoperatively to 41.02 mg/L two weeks postoperatively. Three patients achieved complete recovery within approximately two months with continued wound care and outpatient follow-up. One patient demonstrated clinical improvement but later died due to underlying health conditions unrelated to the procedure. These cases demonstrate that minimally invasive punch and drainage can serve as a safe and effective adjunctive treatment for refractory NP-SSTIs and may represent a practical therapeutic option for patients who respond poorly to antibiotic therapy alone.

## Introduction

In accordance with the 2014 Infectious Diseases Society of America guidelines, skin and soft tissue infections (SSTIs) encompass a spectrum of bacterial infections, ranging from superficial cellulitis to deep necrotizing infections. SSTIs stand out as one of the most prevalent reasons prompting patients to seek both inpatient and outpatient medical treatment [1]. According to previous studies [2,3], SSTIs account for a staggering 14 million outpatient visits and nearly 900,000 inpatient admissions annually in the United States. Non-purulent SSTIs (NP-SSTIs), which include cellulitis, erysipelas, and necrotizing fasciitis without visible abscess formation. Despite the absence of obvious purulent drainage, NP-SSTIs can pose a potentially life-threatening risk if not adequately treated [4].

While antibiotics typically serve as the gold standard for managing SSTIs [5,6], a portion of at least 17% patients, as shown by different studies, may prove refractory to standard treatment [7-9]. We defined refractory cases as those that demonstrate no clinical improvement after five to seven days of appropriate antibiotic therapy, necessitating additional intervention. Antimicrobial treatment failure in adults with SSTIs follows an increase in admission days and mortality. Demonstrated by current studies, patients with high body mass index (BMI) and heart failure are important risk factors for higher antibiotic treatment failure rates [10].

Recognizing that incision and drainage (I&D) represent the gold standard for treating purulent SSTIs, aiming to evacuate inflammatory exudate and eliminate pus collections [5], our study found that the mini-punch and drainage (P&D) procedure also offers considerable advantages in addressing refractory NP-SSTIs. Given the possibility of potential treatment failure and monotonous treatment options of NP-SSTIs, mini-P&D may serve as an effective adjunctive treatment for hard-to-treat NP-SSTIs, especially in patients with compromised health conditions. Our study seeks to provide an insightful overview of our experiences with mini-P&D as an innovative, safe, and effective technique for refractory NP-SSTIs.

## Case presentation

Study population and design

This study was conducted at Kaohsiung Medical University Hospital between December 2021 and December 2023, a tertiary hospital in southern Taiwan. A total of four patients included in this study were all diagnosed with NP-SSTIs but refractory to regular antibiotic treatment. All patients were managed through standard hospital referral pathways, including the emergency department or outpatient clinics, and were referred for surgical evaluation following inadequate response to antibiotic therapy.

Indications for the punch and drainage procedure included persistent local inflammation, progressive pain or swelling, and sustained elevation of inflammatory markers despite medical treatment. All mini-P&D procedures were performed following surgical evaluation, and standard perioperative wound care was provided. Clinical outcomes were assessed using objective measures, including CRP levels, duration of hospitalization, and time to wound healing.

The patient demographics have been presented in Table 1.

**Table 1:** Demographics of patients Note: Case 3 underwent mini-punch and drainage procedure in our outpatient department due to personal preference. Coronary arterial disease (CAD), Type 2 diabetes mellitus (T2DM), osteoarthritis (OA), total knee arthroplasty (TKA), hypertension (HTN), chronic kidney disease (CKD), hepatitis b virus (HBV), white blood cell (WBC), C-reactive protein (CRP)

	Case 1	Case 2	Case 3	Case 4
Age (years)	83	75	99	85
Sex	Male	Male	Female	Female
Underlying diseases	CAD T2DM	OA s/p TKA	HTN CKD HBV carrier	HTN T2DM
Affected site	Right hand	Left leg and Bilateral hands	Bilateral legs	Left leg
Length of stay (days)	7	42	N/A*	12
Time to wound healing (weeks)	8	4	6	8
Follow-up duration (weeks)	8	6	12	8
Follow-up status	Healed	Expired	Recurred twice then healed	Healed
White Blood Cell (x10^9^/L) WBC_Pre_ WBC_Post_	12200 6770	10700 20900	5180 4350	16840 12190
(WBC_Post _- WBC_pre_)/WBC_pre_ *100 (%)	-45%	+95%	-16%	-28%
C-reactive protein (mg/L) CRP_Pre_ CRP_Post_	96.72 6.85	187.83 123.86	45.74 11.19	207.79 22.19
(CRP_Post _- CRP_pre_)/CRP_pre_ *100 (%)	-93%	-34%	-76%	-89%

Operation method

After discussion with the patients, mini-P&D procedures were performed by a single well-trained plastic surgeon. Three patients underwent the mini-P&D procedure in the operating room, and one patient received the mini-P&D procedure in the clinic due to her family's preferences. Multiple circular incisions were created using a skin biopsy punch, which facilitated drainage of inflammatory exudate from the infection sites, including interstitial fluid, leukocytes (particularly neutrophils), pro-inflammatory cytokines (e.g., interleukin-6 (IL-6) and tumor necrosis factor-α (TNF-α)), and tissue debris. Each wound diameter was about 2 to 6 mm, resulting in lower surgical risk and acceptable postoperative scar appearance. Penrose drains were placed in deeper cavities as necessary and secured with 4-0 nylon sutures.

Patient characteristics

Among all four patients, the average age was 86.8 years (ranging from 75 to 99). Underlying comorbidities included hypertension, type 2 diabetes mellitus, coronary artery disease, and chronic kidney disease. The mean preoperative C-reactive protein (CRP) level was 134.52 mg/L (ranging from 45.74 to 207.79 mg/L). The mean CRP level decreased to 41.02 mg/L (ranging from 6.85 to 123.86 mg/L) two weeks after the procedures. All patients expressed satisfaction with the clinical outcomes, and the postoperative scar appearance was acceptable. All patients’ clinical conditions and blood tests showed significant improvements two weeks after the surgery and then reached complete healing in two months. The follow-up duration ranged from 6 to 12 weeks (mean: 8.5 weeks). Two patients achieved complete clinical resolution without recurrence, while one patient experienced two episodes of cellulitis recurrence within three months of follow-up. The remaining patient unfortunately expired due to poor overall health conditions. No significant adverse events were noticed in these patients.

Case 1

**Figure 1:**
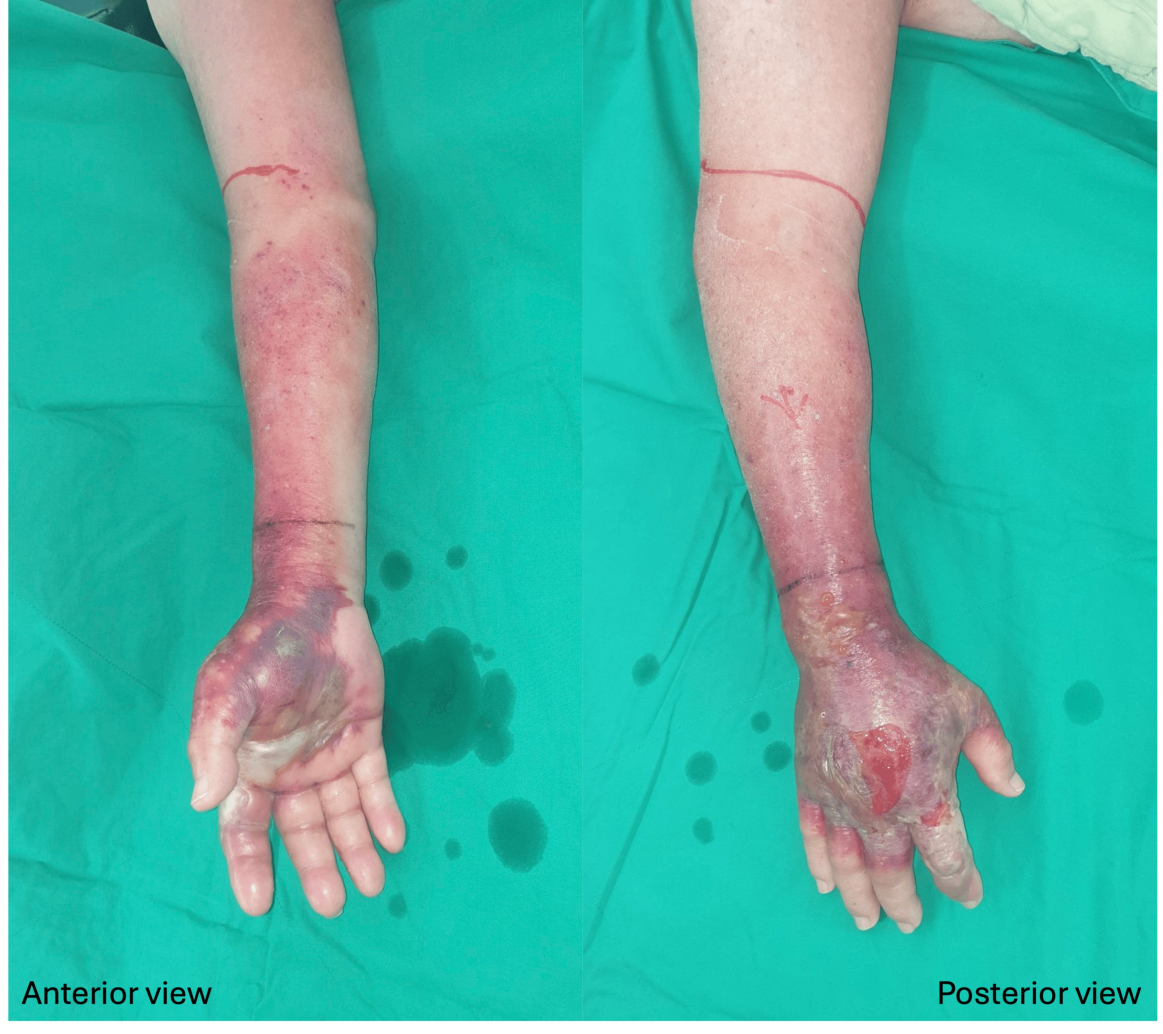
Non-purulent skin and soft tissue infection was diagnosed; however, the clinical condition continued to deteriorate despite antibiotic treatment.

An 83-year-old man with a history of coronary artery disease and type 2 diabetes mellitus presented with progressive redness, swelling, and tenderness of the right hand. Antibiotic treatment was prescribed for five days at a local hospital, but his clinical condition continued to deteriorate (Figure 1).

Consequently, he sought help at our emergency department (ED). Computed tomography (CT) scan images revealed increased infiltration at the subcutaneous layer and deep fascia over the right hand and wrist. Moderate NP-SSTI was diagnosed. 

**Figure 2:**
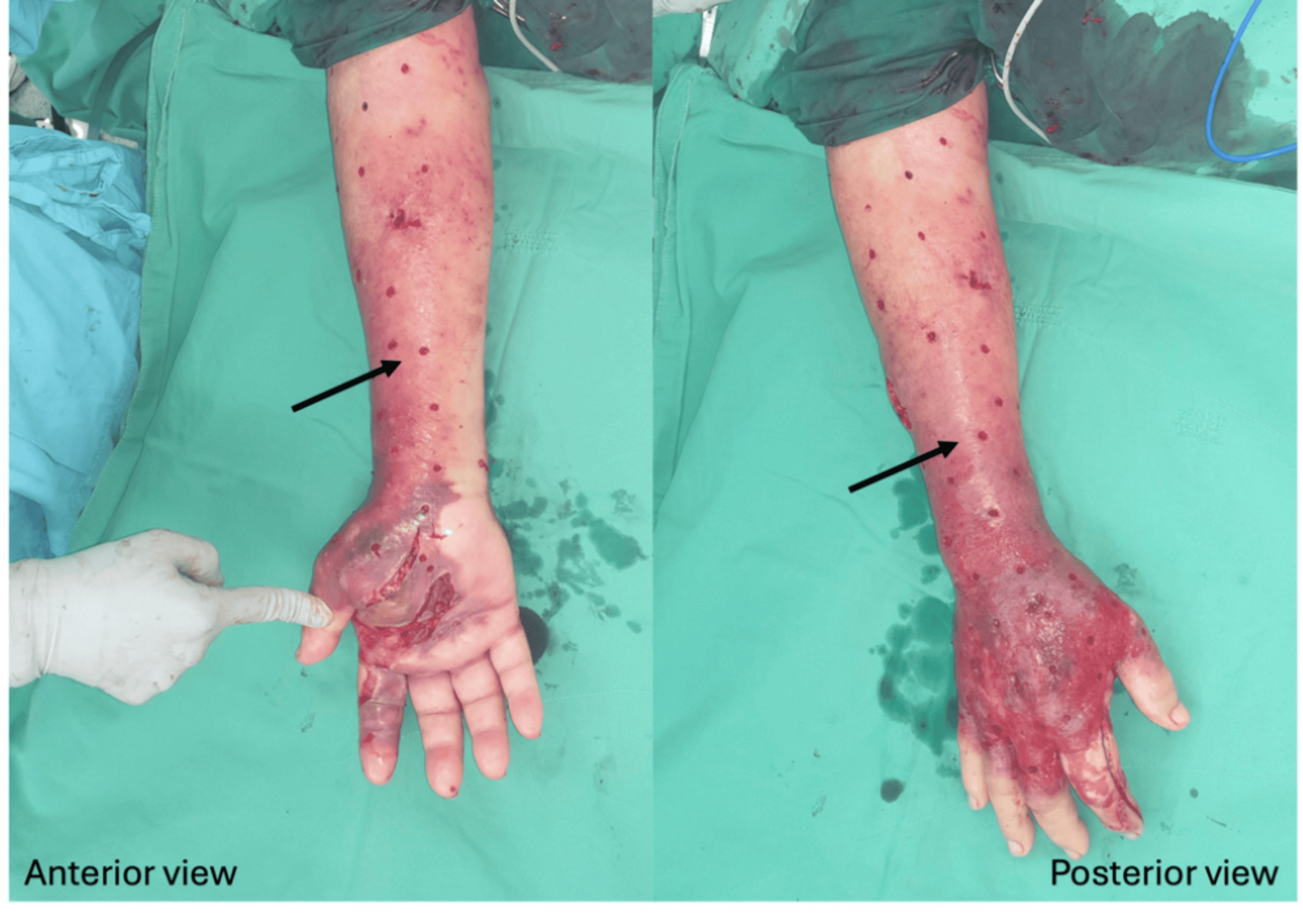
The mini-P&D procedure was performed on the patient’s right forearm, and fasciotomies were also performed on the left index finger and palm by an experienced surgeon (arrow indicates the punch and drainage site). mini-P&D, mini-punch and drainage

Following surgical consultation, right-hand mini-P&D procedure was suggested and performed (Figure 2).

**Figure 3:**
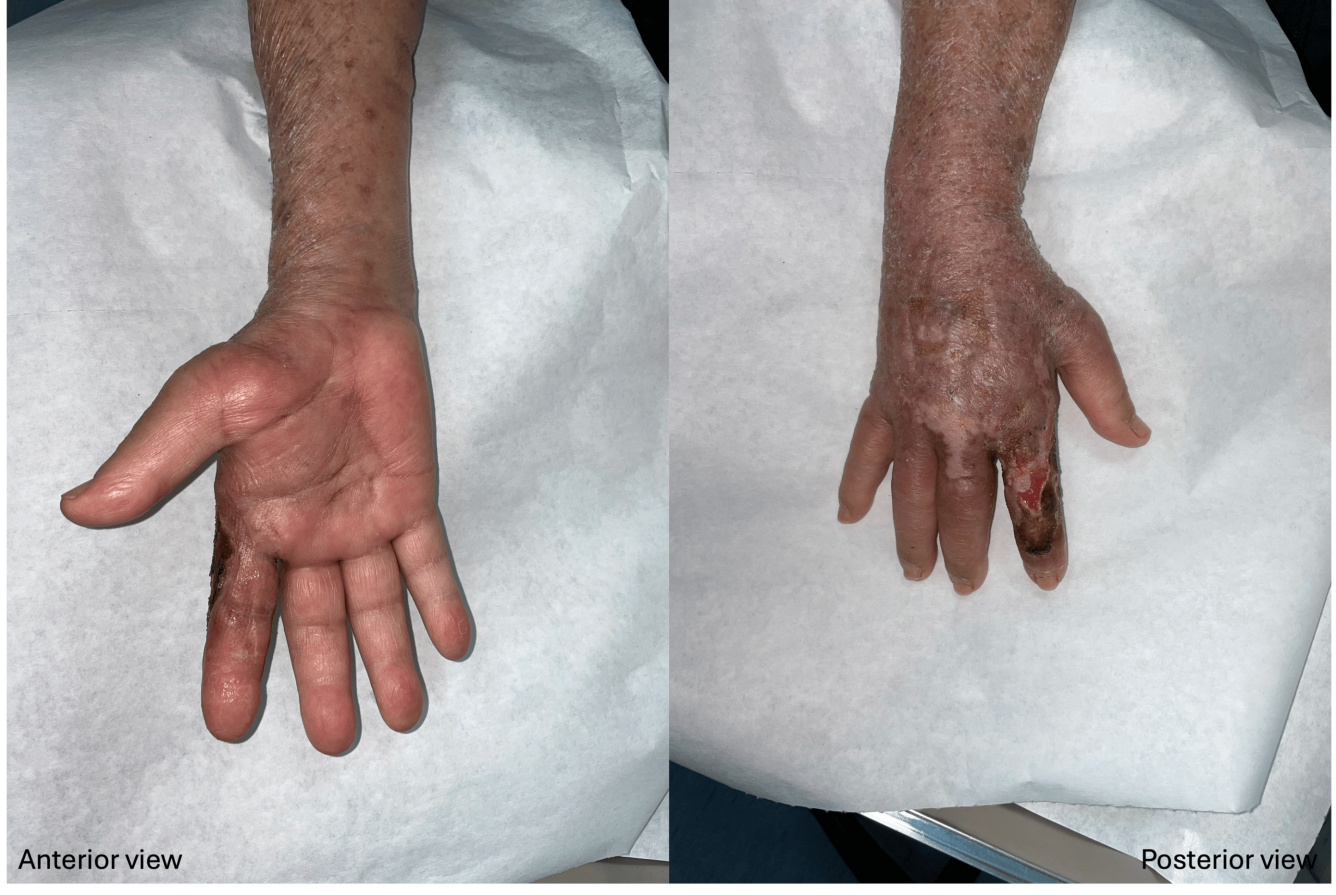
The wound was stable, and most of it had healed following surgical treatment; however, the patient was lost to follow-up after two months of wound care.

Subsequent blood tests showed a gradual decrease in both WBC and CRP levels, indicating the efficacy of the surgical procedure. The patient's clinical condition significantly improved, and he was discharged after seven days of diligent wound care. The wound had almost healed after two months of outpatient department (OPD) follow-up (Figure 3).

However, the patient was subsequently lost to follow-up.

Case 2

A 75-year-old male with a history of osteoarthritis and a status post total knee arthroplasty experienced intermittent fever and dyspnea for a few days, prompting him to seek help at our ED. Upon arrival, hypotension, tachycardia, and desaturation were noticed. The physical examination revealed bilateral crackles and pitting edema in both lower legs. Blood tests indicated leukocytosis, high CRP levels, and metabolic acidosis. Chest X-ray revealed cardiomegaly and pulmonary congestion. CT scan images of the bilateral lower legs showed increased interstitial edema and mild loculated effusion in both knees. An orthopedist performed arthrocentesis to address the bilateral knee effusion, and pus-like content was aspirated and sent for culture. He was subsequently admitted to the Medical Intensive Care Unit to correct his shock status and subsequent complications.

**Figure 4:**
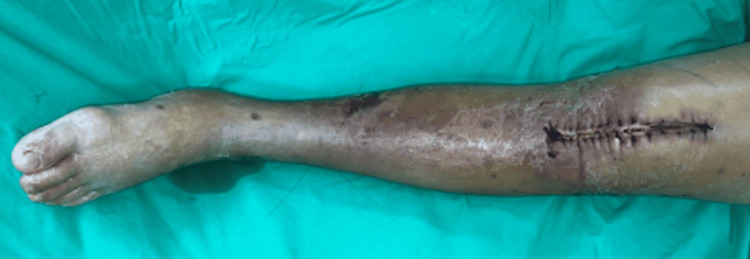
Local fasciitis of the left leg was diagnosed; therefore, a mini-P&D procedure was recommended. mini-P&D, mini-punch and drainage

Despite broad antibiotic treatment, the patient's hemodynamics remained unstable. Subsequent blood culture showed bacteremia. The orthopedist removed the prosthesis and excised necrotic tissue around both knees. Follow-up CT scan images still revealed left leg local fasciitis but persistent fluid accumulation over bilateral knees. A diagnosis of severe NP-SSTI was made, prompting a referral to a surgeon for further treatment planning (Figure 4).

**Figure 5:**
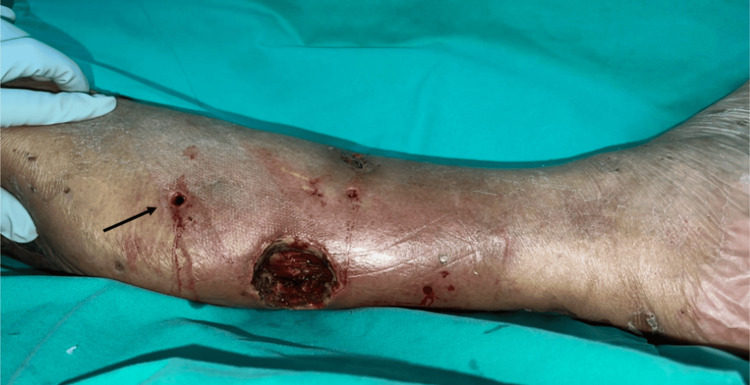
The mini-P&D procedure was performed on the patient’s left leg (arrow indicates the punch and drainage site). mini-P&D, mini-punch and drainage

Mini-P&D procedure was first performed over his left leg (Figure 5).

**Figure 6:**
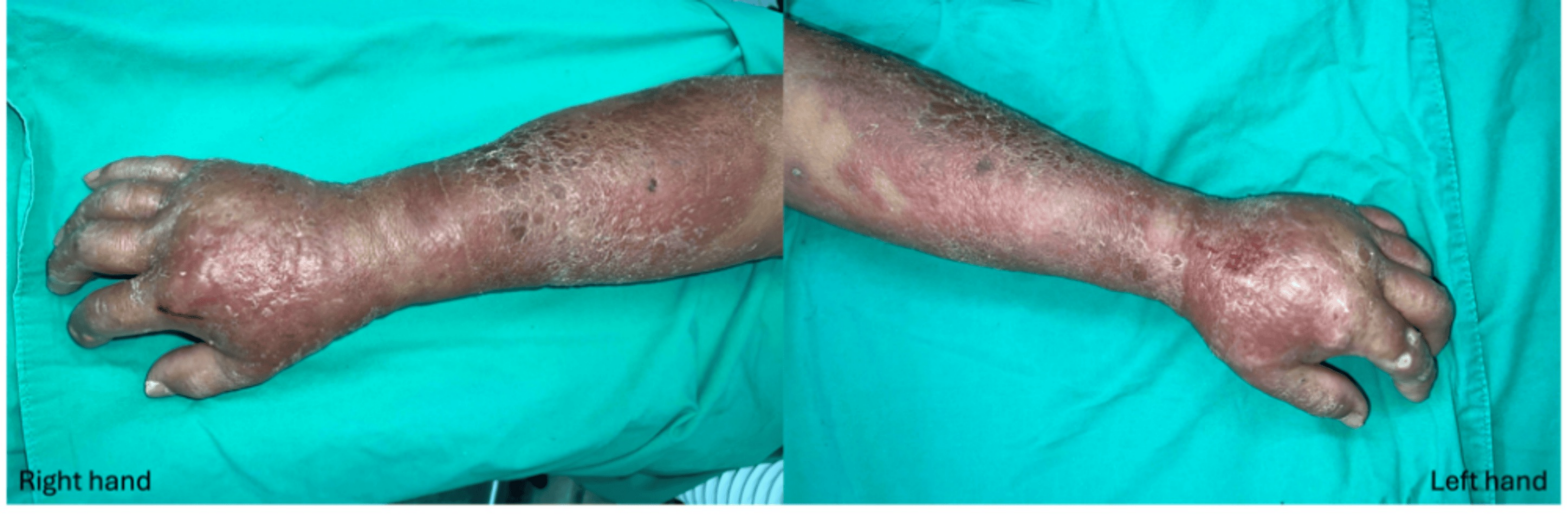
Progressive redness, swelling, and tenderness were noticed over the patient's bilateral forearms.

**Figure 7:**
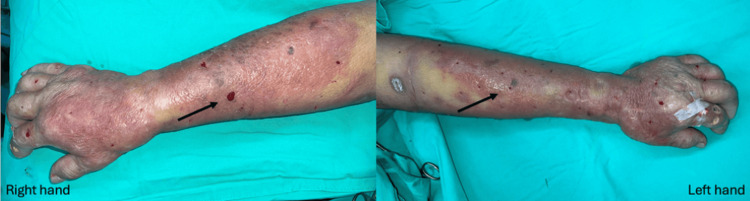
Given the patient’s poor clinical condition, the mini-P&D procedure was performed on both hands (arrow indicates the punch and drainage sites). mini-P&D, mini-punch and drainage

Due to progressive redness, swelling, and tenderness over the patient’s bilateral forearms (Figure 6), mini-P&D procedures were also performed (Figure 7).

**Figure 8:**
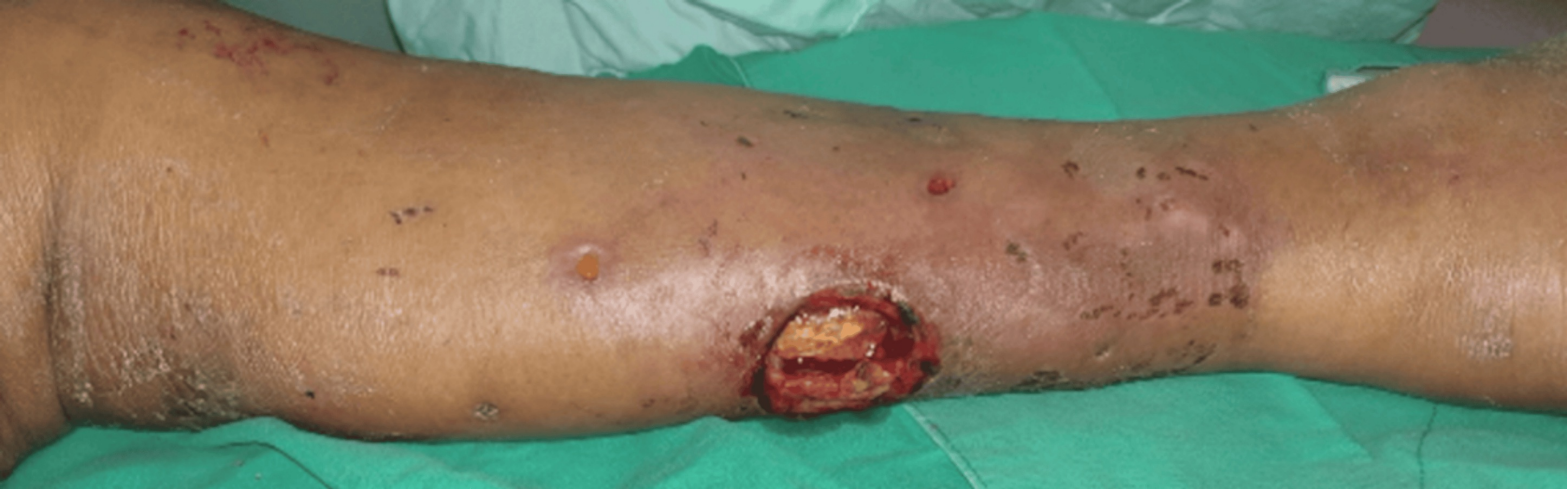
The patient’s clinical condition was finally brought under control after surgical treatment, and the wound improved following one month of diligent wound care.

**Figure 9:**
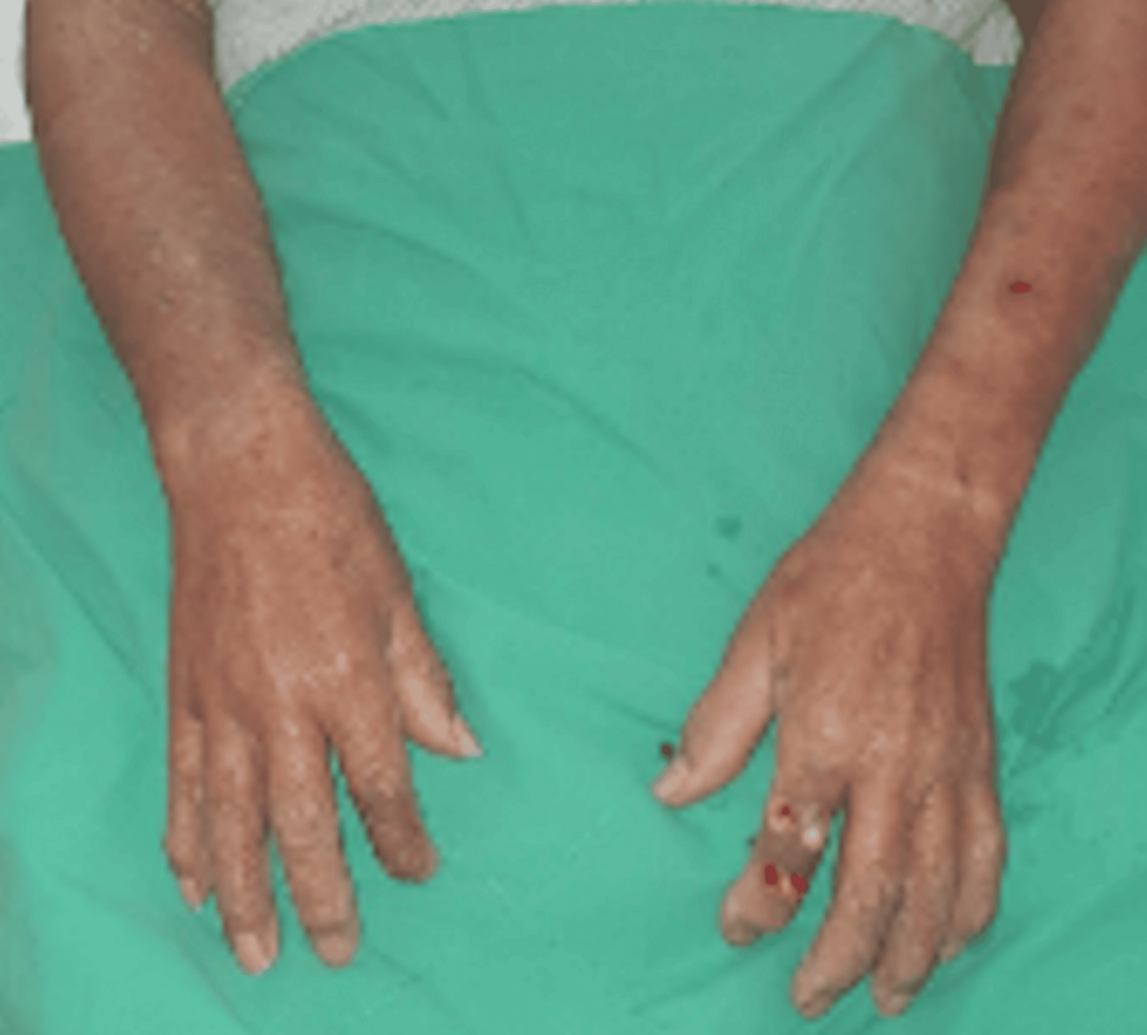
The patient’s clinical condition was under control after surgical treatment, and the upper extremity wound healed completely after one month of diligent wound care.

The patient’s clinical condition was finally brought under control after surgical intervention, and the wounds had successfully healed one month later (Figures 8-9).

Due to the poor prognosis associated with his underlying health conditions, the patient was transferred to the hospice ward two weeks after surgery and passed away one month later.

Case 3

**Figure 10:**
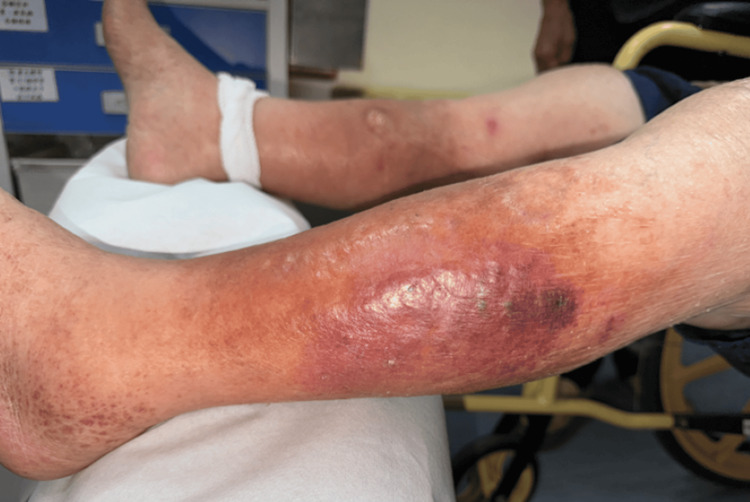
Progressive redness, swelling, and tenderness were noted after a few days of antibiotic treatment.

A 99-year-old female with a history of hypertension, chronic renal disease, and chronic hepatitis B infection experienced bilateral leg swelling for a few days. Mild NP-SSTI was diagnosed. She initially sought medical help at a local clinic and was later transferred to our OPD for wound management (Figure 10).

**Figure 11:**
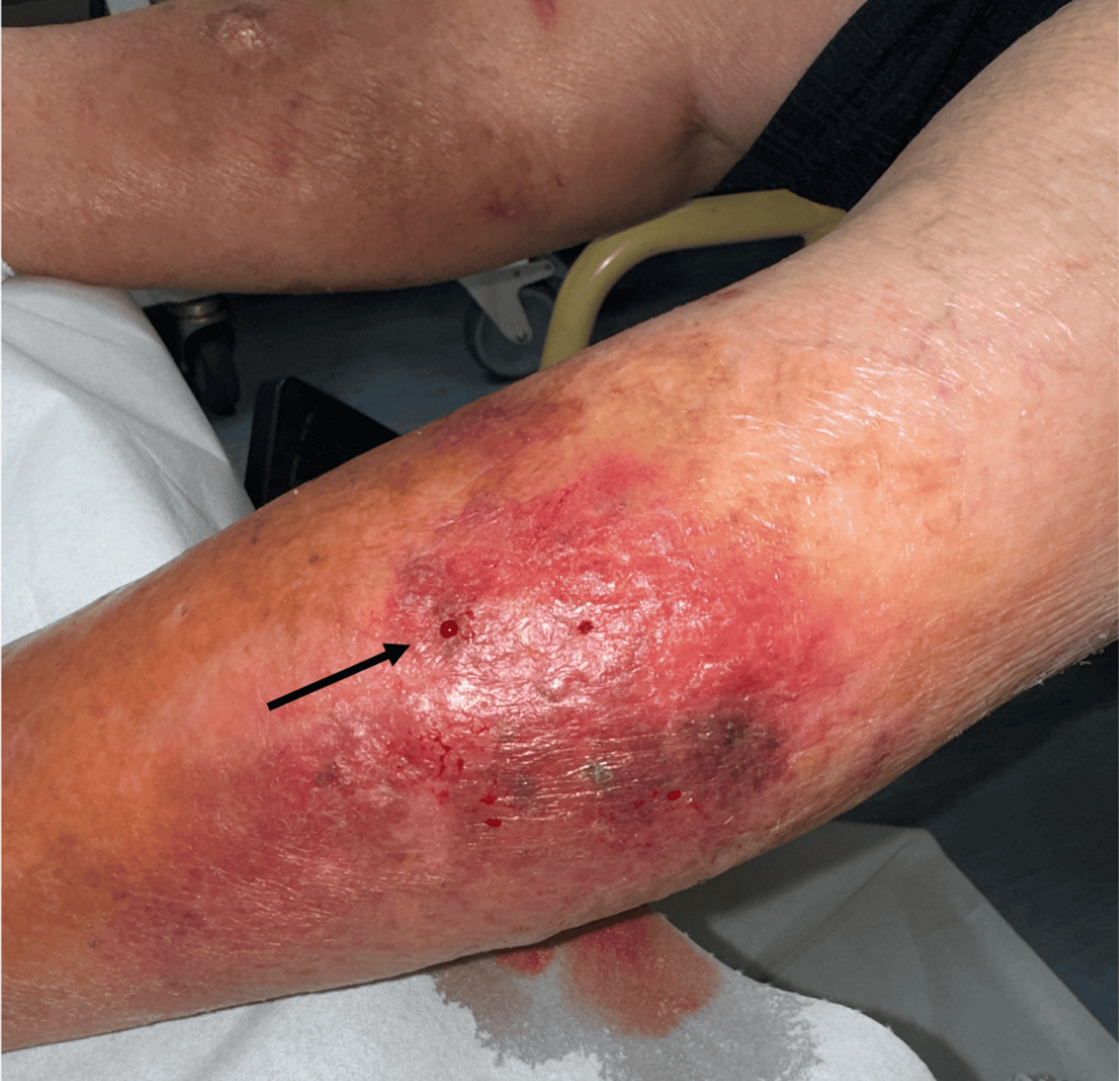
The mini-P&D procedure was recommended and performed on the patient’s bilateral legs (arrow indicates the punch and drainage sites). mini-P&D, mini-punch and drainage

Antibiotics were prescribed initially, but the clinical condition failed to improve. Therefore, mini-P&D procedure was performed in the OPD (Figure 11).

**Figure 12:**
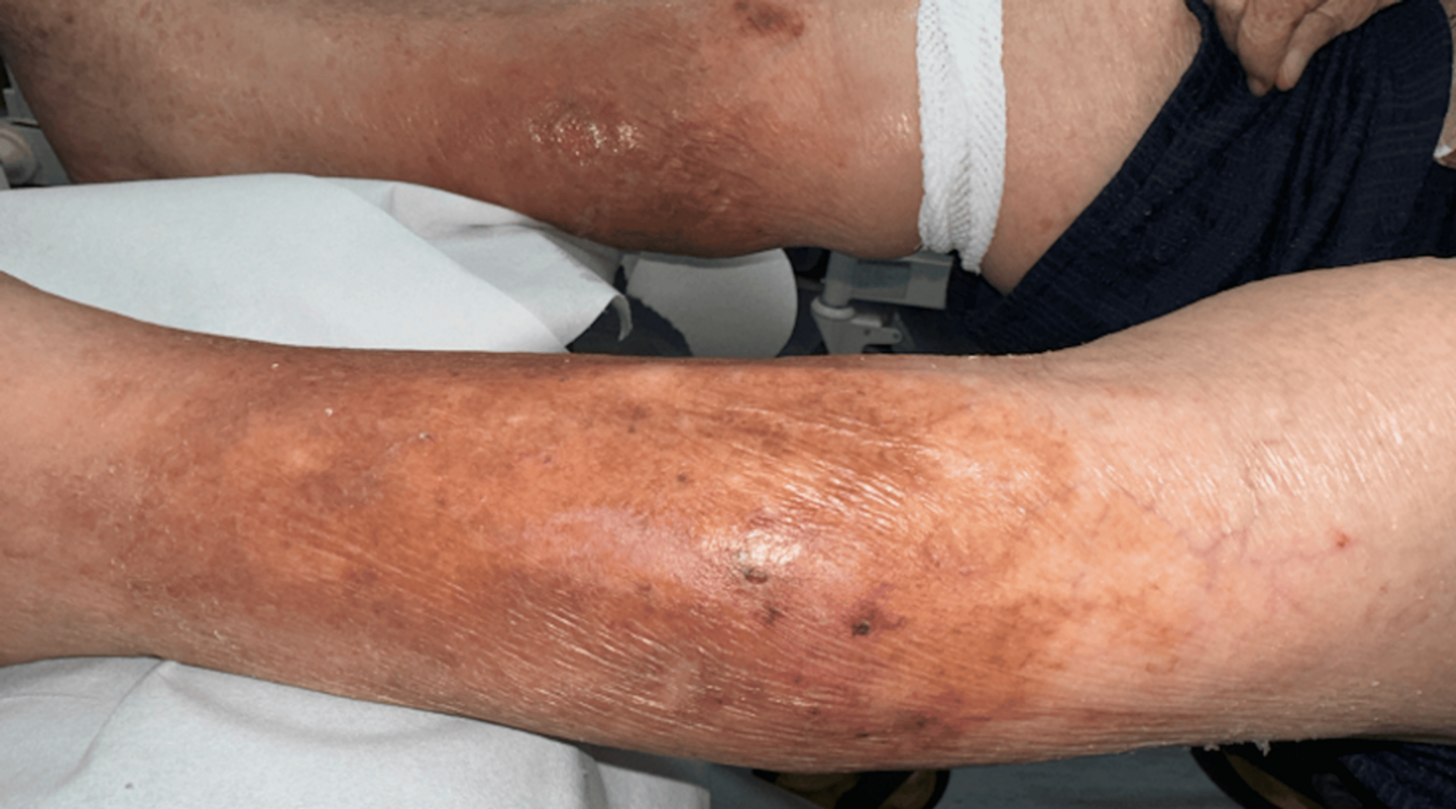
The wound healed completely after surgical treatment and diligent wound care for one month.

Subsequent blood tests showed a gradual decrease in CRP levels, indicating the efficacy of the surgical procedure. The swelling in her bilateral legs was successfully controlled after six weeks of diligent wound care (Figure 12).

However, the patient experienced two recurrences within three months of follow-up. The same surgical treatment was performed due to refractoriness to antibiotics, and she eventually healed completely.

Case 4

**Figure 13:**
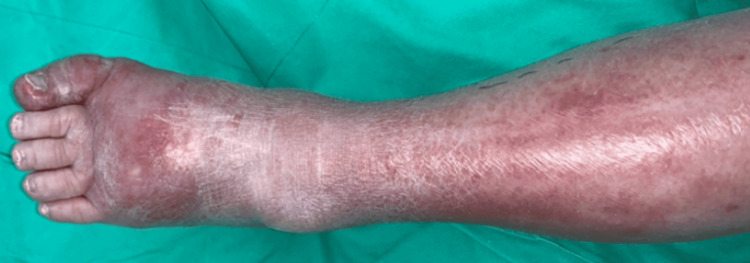
Progressive redness, swelling, and tenderness of the left leg were observed after five days of antibiotic treatment.

An 85-year-old female with a history of hypertension and type 2 diabetes mellitus experienced left leg swelling and pain for five days. Antibiotic treatment was prescribed for five days, but her clinical condition continued to deteriorate. Consequently, she sought help at our ED. CT scan images revealed subcutaneous infiltration over the left lower leg. The patient was diagnosed with moderate NP-SSTI (Figure 13).

**Figure 14:**
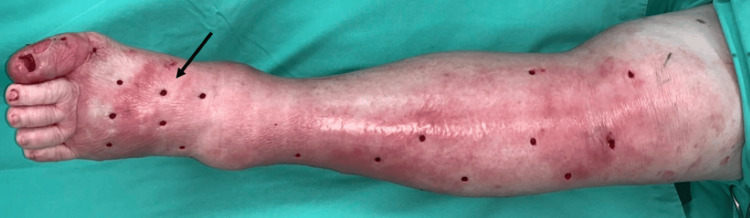
The mini-P&D procedure was recommended and performed on the patient’s left leg (arrow indicates the punch and drainage site). mini-P&D, mini-punch and drainage

Following surgical consultation, mini-P&D was suggested and performed on the patient (Figure 14).

**Figure 15:**
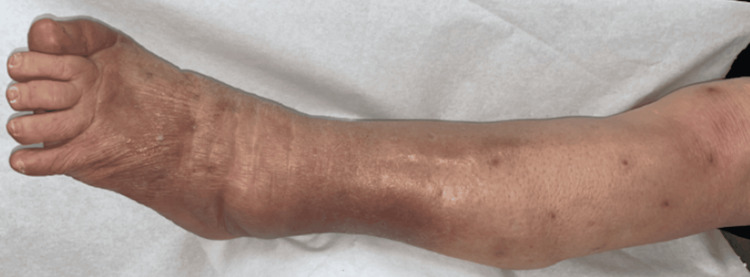
The wound healed completely after surgical treatment and diligent wound care for two months.

Subsequent blood tests showed a gradual decrease in CRP levels, indicating the efficacy of the surgical procedure. The patient's clinical condition significantly improved, and she was discharged after two weeks of diligent wound care. The wound completely healed after two months of surgical treatment (Figure 15).

## Discussion

Antibiotic treatment failure rates in patients with SSTIs range from 6% to 37%, as demonstrated in previous studies [11]. Apart from further incision and drainage to address complicated purulent wounds, we currently have no alternative treatment options for patients with hard-to-treat NP-SSTIs, except for shifting to broad-spectrum antibiotics and performing extensive fasciotomy in patients with necrotizing fasciitis according to current guidelines [5]. The failure to treat complex clinical situations eventually leads to life-threatening conditions with significantly elevated amputation and mortality rates, requiring admission to the intensive care unit [12-15]. Therefore, it is of utmost importance to develop a new technique for these challenging patients with NP-SSTIs. 

The results of this study show that mini-P&D performed on patients with NP-SSTIs who are refractory to regular antibiotic therapy had good clinical outcomes. It has been demonstrated that our method efficiently ameliorates patients' clinical status. The visual analog scale (VAS) score ranged from 0 to 2, with a median of 2 on days 1-3 following surgery. No deterioration of necrotizing fasciitis was observed in our study, which is a more severe clinical condition. 

Traditional extensive necrotic tissue debridement typically results in large-area tissue trauma, leading to prolonged hospitalization and a higher complication rate with vital structure exposure. In contrast, the surgical wound size of mini-P&D is approximately 2-6 mm for each circular incision. This approach preserves more viable soft tissue, reducing the risk of introducing additional pathogens into the wound and preventing the hematogenous spread of infection and secondary contamination usually caused by extensive incisions. The mini-P&D procedure minimizes the risk of premature closure by creating defined circular incisions rather than a linear scar, eliminating the chance of closely approximated wound margins [16]. This allows inflammatory contents to continue draining freely and completely. A similar punch technique has been proven effective and practical in patients with abscesses [16]. However, this is the first study to discuss the prospect of applying it to patients with difficult-to-treat NP-SSTIs.

In our study, punch and drainage have demonstrated superior patient outcomes in terms of post-procedural pain and discomfort, as inflammatory cytokines can be thoroughly drained. No adverse events were observed in all four included patients. The smaller wound size may contribute to quicker healing, reducing the overall treatment duration and complications. It is worth mentioning that the physician’s experience is also of utmost importance in deciding the timing of the performed technique. According to the latest clinical follow-up of the patients, our technique yielded acceptable postoperative scar appearance and postoperative satisfaction in all cases.

It is important to acknowledge the limitations of this study. This was a single-center case series (*N* = 4), without randomization or a control group. The small sample size and absence of comparative data limit the generalizability of the findings. There is also a potential for selection bias, as patient inclusion was not blinded or randomized. Readers are therefore advised to interpret the findings with caution.

Moreover, while the mini-P&D technique demonstrated clinical improvement in these cases, it may not be suitable for all types of NP-SSTIs. In particular, for patients diagnosed with necrotizing fasciitis, the limited excisional area from the punch wound is unlikely to provide adequate debridement [17]. In such cases, mini-P&D should be considered an adjunctive tool alongside traditional surgical debridement. This combination may help reduce wound burden while preserving effective infection control.

Finally, although favorable trends were observed in CRP reduction, pain relief, and wound healing, the short follow-up period and lack of standardized outcome measures warrant further investigation. This study represents our preliminary experience. Future studies with larger sample sizes, multicenter collaboration, and comparative designs are needed to validate the clinical utility and reproducibility of the mini-P&D approach and to develop standardized treatment protocols for refractory NP-SSTIs.

This technique is not limited to a single specialty and may be adopted by appropriately trained clinicians across surgical disciplines as part of a multidisciplinary approach to refractory NP-SSTIs.

## Conclusions

Our study demonstrates that mini-P&D represents a promising treatment option for patients with refractory NP-SSTIs. This approach achieves significant clinical improvement by directly reducing inflammatory substances while minimizing the risk of complications by avoiding harm to surrounding soft tissues and exposure of underlying vital structures. We suggest that clinicians consider this procedure as a safe, effective, and valuable treatment option for managing NP-SSTIs, particularly when antibiotics alone prove stagnated.
